# Single‐stage orthognathic correction of campylorrhinus lateralis in foals using transection and fixation: Surgical description and outcome of four horses

**DOI:** 10.1002/evj.70227

**Published:** 2026-07-18

**Authors:** Helio Vasco, Jim Schumacher, Jack Easley

**Affiliations:** ^1^ Kentucky Equine Veterinary Dental Specialists Versailles Kentucky USA; ^2^ College of Veterinary Medicine, Department of Large Animal Clinical Sciences The University of Tennessee Knoxville Tennessee USA

**Keywords:** class 4 malocclusion, horse, maxillofacial surgery, orthognathic surgery, transection and fixation, wry nose

## Abstract

**Background:**

Campylorrhinus lateralis (‘wry nose’) is a rare congenital maxillofacial deformity of foals resulting in compromise of the airway and severe class 4 malocclusion. Various surgical techniques to correct the deformity have been described, but contemporary data on single‐stage transection and fixation, including use of a rib graft, and long‐term functional and cosmetic outcomes remain underreported.

**Objectives:**

To describe the transection‐and‐fixation technique and perioperative management used to correct wry nose in horses, and to report postoperative complications and long‐term outcomes of horses undergoing this treatment.

**Study Design:**

Retrospective case series.

**Methods:**

Wry nose of four horses was corrected by using a single‐stage transection‐and‐fixation technique. Pre‐operative assessment included physical, oral, and radiographic examinations, and when available, CT imaging. The surgical procedure involved temporary tracheostomy and laryngotomy, resection of the nasal septum, osteotomy and fixation of the nasal bones, and orthognathic transection and fixation of the premaxillae and maxillae, with bone grafting and alar fold resection performed when indicated. The outcome of horses with complete records and ≥12 months follow‐up is reported using descriptive statistics.

**Results:**

All four horses were presented with respiratory difficulty. The transected premaxillae were stabilised with pins and wires, and the transected nasal bones were stabilised with plates/screws; only one horse received a rib graft. All horses developed a plate‐associated infection, and one horse developed an oronasal fistula. At the time of follow‐up, all grazed and masticated normally, maintained good body condition, and owners were satisfied with the functional and cosmetic outcome.

**Main Limitations:**

Small sample size, retrospective design.

**Conclusions:**

Correcting wry nose of four foals using a single‐stage transection‐and‐fixation procedure achieved satisfactory long‐term outcomes. Respiratory function of all horses improved, and all horses grazed and masticated normally. Three horses were able to perform their intended athletic endeavour. All owners reported satisfaction with the functional and cosmetic outcome.

## 1 INTRODUCTION

Campylorrhinus lateralis, or wry nose, is a rare congenital deformity of the maxillofacial bones characterised by lateral and often rotational deviation of the premaxillae and maxillae, nasal bones, vomer bone, and nasal septum, resulting in marked facial asymmetry and class 4 malocclusion.^1^, ^2^, ^3^, ^4^, ^5^, ^6^, ^7^ The aetiology appears to be multifactorial and incompletely defined.^8^, ^9^

Affected foals usually exhibit obstruction of nasal airflow, abnormal respiratory noise, difficulty suckling, and impaired prehension of forage.^1^, ^10^ Horses with mild deformities may be managed conservatively with routine dental care, whereas moderately to severely affected horses, particularly those that have associated respiratory difficulty, are generally euthanised or referred for surgical correction, which primarily aims to re‐establish patency of the airway and correct dental malocclusion, rather than achieve perfect facial symmetry.^1^, ^2^, ^5^, ^7^, ^10^

Several surgical approaches have been described to correct wry nose, including the technique described by Valdez et al., a staged premaxillary osteotomy stabilised with a rib graft and Steinmann pins combined with resection of the deviated portion of the nasal septum^1^; use of an external fixator^3^, ^4^; and distraction osteogenesis.^11^, ^12^ Schumacher and colleagues^2^, ^13^ refined Valdez's approach^1^ into a single‐stage, orthognathic procedure, termed the ‘transection‐and‐fixation technique’, which combines osteotomy of the premaxillae/maxillae and nasal bones with internal fixation (typically using bone plates and screws to stabilise the nasal bones and Steinmann pins and Kirschner wires to stabilise the premaxillae) and a more extensive resection of the nasal septum.

The aim of this retrospective case series is to describe the surgical techniques used to correct wry nose of four weanlings and the perioperative management, postoperative complications, and long‐term outcome of these horses.

## 2 MATERIALS AND METHODS

### 2.1 Study design and data description

This retrospective series describes four horses with wry nose (campylorrhinus lateralis) that underwent surgical correction using a transection‐and‐fixation technique described by Schumacher et al. (2008).^2^ Only descriptive statistics were used because of the small number of horses. Continuous variables (e.g., age at time of surgery and time of follow‐up) were summarised as medians and ranges, and categorical variables (e.g., the presence or absence of specific complications [Table 1]) were summarised as counts and percentages.

**TABLE 1:** Postoperative complications of four horses after surgical correction of wry nose.

Case	Premature extrusion of the pin and wires	Infection at implanted plates and screws	Collapse of tracheal rings	Oronasal fistula	Suture dehiscence of the oral incisions	Class 3 malocclusion
01	No	Yes	No	Yes	Yes	Yes
02	No	Yes	Yes	No	Yes	No
03	No	Yes	No	No	Yes	No
04	Yes	Yes	No	No	No	No

### 2.2 Study population

Horses eligible for inclusion were those referred to two of the authors (JS and JE) between 2022 and 2024 for surgical treatment for wry nose. All cases meeting the inclusion criteria during the study period were included. Inclusion criteria were: (1) use of the technique described by Schumacher and colleagues,^2^ (2) complete medical records, and (3) a minimum postoperative follow‐up of 12 months. Data extracted for each horse included signalment (i.e., breed, sex, and age), presenting clinical signs, findings during physical examination, radiographic and/or computed tomographic findings, and detailed intraoperative and postoperative clinical data. Cases included in this case series were also part of a separate retrospective survey‐based study evaluating surgeon‐ and owner‐reported long‐term outcomes.^14^

### 2.3 Pre‐operative evaluation and radiological diagnosis

Each horse received a complete physical examination, including oral examination, with specific attention given to identifying concomitant congenital and respiratory abnormalities. The degree and planes of maxillofacial deviation were assessed clinically and radiographically using standard radiographic projections of the skull; where available, computed tomography (CT) was used to characterise the three‐dimensional deformity of the facial bones and nasal septum and to assist planning of sites of osteotomy. Pre‐anaesthetic haematology and serum biochemical analyses were performed at the discretion of the attending clinicians. Routine odontoplasty was performed before surgery to reduce sharp enamel points on the cheek teeth using standard dental instrumentation, and the incisors were levelled to restore a near‐normal occlusal angle and to facilitate realignment of incisors after orthognathic correction.

### 2.4 Patient anaesthesia and analgesia

All horses received gentamicin sulphate (6.6 mg/kg IV; Gentamicin Injection, USP; Akorn Pharmaceuticals) and a non‐steroidal anti‐inflammatory drug, flunixin meglumine (1.1 mg/kg IV; Banamine®, Merck Animal Health), as a single dose, 40–60 min prior to surgery.

Horses were premedicated with xylazine hydrochloride (0.6–1.1 mg/kg IV; Rompun®, Elanco Animal Health) in combination with butorphanol tartrate (0.01–0.02 mg/kg IV; Torbugesic®, Zoetis). Anaesthesia was induced with ketamine hydrochloride (2.2 mg/kg IV; Ketaset®, Zoetis Inc.) and diazepam (0.05 mg/kg IV; Akorn Pharmaceuticals).

After induction of anaesthesia, the horse was placed in dorsal recumbency for performing a laryngotomy and temporary tracheotomy, and anaesthesia was maintained with intravenous infusion of a combination of ketamine (1000 mg/500 mL), xylazine (500 mg/500 mL), and guaifenesin (50 mg/mL), administered at 1.0–2.0 mL/kg/h, and adjusted to effect.

After performing the tracheostomy and laryngotomy, the horse was placed in lateral recumbency, intravenously administered anaesthesia was discontinued, and the horse was administered sevoflurane (SevoFlo®, Zoetis Inc.) delivered in oxygen through a large animal circle breathing system. Ventilation was spontaneous, but intermittent manual positive‐pressure ventilation was administered as required.

The right and left infraorbital nerves were anaesthetised with mepivacaine hydrochloride (Carbocaine®‐V Sterile Aqueous Solution, Zoetis Inc.) using a previously described technique^15^, ^16^ to desensitise the premaxillae and maxillae.

### 2.5 Surgical technique: Transection and fixation

The procedures were performed during a single anaesthetic episode in five phases, adapted from previously described techniques for orthognathic correction of wry nose.^2^, ^7^, ^13^

#### 2.5.1 Laryngotomy and temporary tracheostomy

With the horse anaesthetised and in dorsal recumbency, the ventral aspect of the neck was prepared for surgery and draped. A 6‐ to 8‐cm cutaneous incision was made on the midline at the junction of the proximal and middle thirds of the cervical portion of the trachea. The incision extended through the subcutaneous tissue and the paired sternothyrohyoideus muscles to expose 3 to 4 tracheal rings. A transverse incision was made through the annular ligament between two rings, and a cuffed endotracheal tube was inserted into the lumen of the trachea.

A cutaneous incision that extended from the rostral aspect of the thyroid cartilage to just caudal to the cricoid cartilage was made over the ventral aspect of the larynx. This incision extended through the subcutaneous tissue and the paired sternothyrohyoideus muscles to expose the ventral aspect of the larynx. The cricothyroid ligament and underlying mucosa were incised longitudinally to expose the lumen of the larynx and allow digital access to the nasopharynx for positioning obstetrical wires for excision of the nasal septum (Figure 1).

**FIGURE 1:**
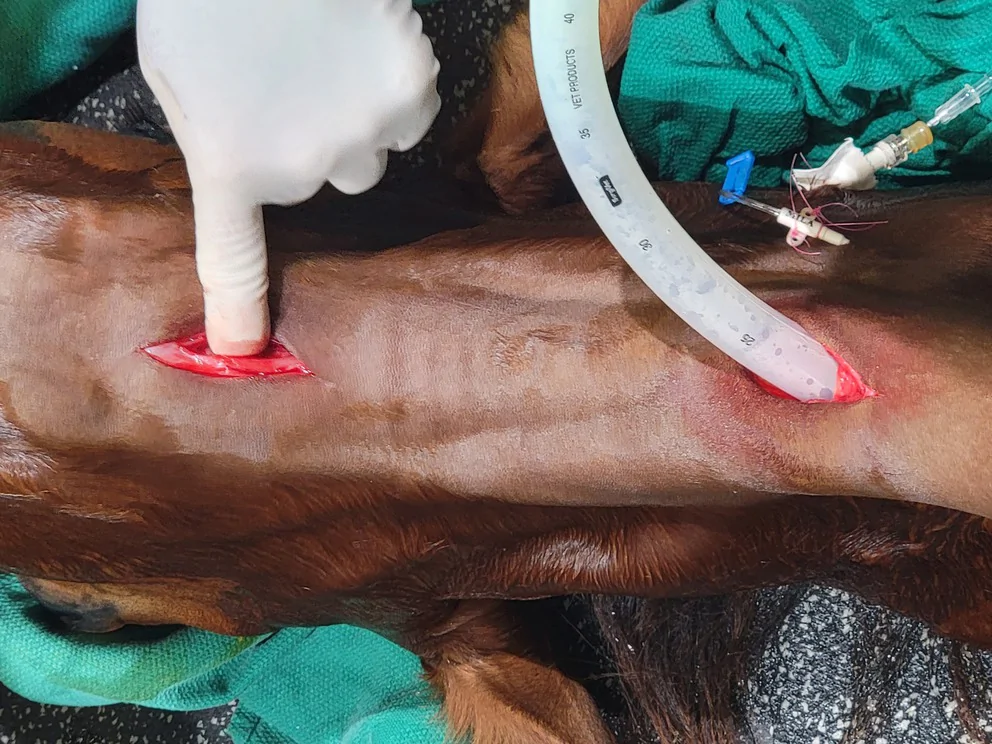
The laryngotomy for positioning wires for excising the nasal septum and temporary tracheostomy for delivering a gas anaesthetic during surgery. The sites of laryngotomy (finger) and temporary tracheostomy (endotracheal tube exiting the trachea) are shown after placement of the endotracheal tube.

#### 2.5.2 Harvesting the rib graft

With the horse in lateral recumbency and the concave side of the deformity uppermost, a 10‐ to 12‐cm cutaneous incision was made over a rib located in the middle of the caudal half of the thorax. The incision began at the costochondral junction and extended dorsally along the long axis of the rib. The incision was deepened through the subcutaneous tissue, cutaneous trunci muscle, and periosteum.

The periosteum was elevated around the entire circumference of the rib with a periosteal elevator, taking care not to penetrate the pleura. A 2‐ to 4‐cm segment of rib was then removed by using an oscillating saw. The segment of rib was wrapped in sterile gauze moistened with isotonic saline solution and blood and stored on the surgical table.

The periosteum, panniculus muscle, and subcutaneous tissue were closed separately with absorbable suture using a simple‐continuous pattern, and the skin was stapled. A stent bandage was sutured over the sutured incision.

#### 2.5.3 Resection of the nasal septum

The nasal septum was excised, with the horse in lateral recumbency, after preparing the sites of incision for surgery, using a modification of the three‐wire technique described by others.^17^, ^18^, ^19^ To position loops of obstetrical wire to create the ventral, dorsal, and caudal incisions for removing the nasal septum, one end of a 1‐m long section of obstetrical wire, covered with a 9‐ or 10‐Fr dog urinary catheter to protect the respiratory mucosa, was passed through the right ventral nasal meatus and the other end, also covered with a dog catheter, through the left ventral nasal meatus. The ends were passed into the nasopharynx and guided through the laryngotomy using a finger inserted into the nasopharynx through the laryngotomy. The catheters were removed, the ends of the wire tied together, and the knot covered with tape. The knot was pulled back into the nasopharynx and out through the right or left nasal passage, seating the loop around the ventrocaudal border of the septum.

The loop of wire used to incise the dorsal border of the septum and that used to create the caudal septal incision were positioned through a portal into the nasal cavity created on the dorsal midline, immediately rostral to the conchofrontal sinuses. The rostral extent of the conchofrontal sinuses was determined radiographically by using skin staples as markers. To create the portal, the nasal bones were exposed through a 4‐ to 5‐cm longitudinal cutaneous incision created on the dorsal midline. A 9.5‐ or 12.7‐mm circular incision was created in the nasal bones with a trephine to expose the underlying parietal cartilage. No attempt was made to preserve the periosteum of the nasal bones. After excising a circular plug of bone, a 10‐mm longitudinal incision was created in the parietal cartilage on each side of the midline to expose the right and left nasal passages through which the loops of obstetrical wire were inserted for the dorsal and caudal septal incisions.

To position a loop of obstetrical wire for the dorsal septal incision, each end of a loop of obstetrical wire, ensheathed in a catheter, was inserted through the trephine portal into the nasal cavity. One end was inserted into the right dorsal nasal meatus, and the other into the left dorsal nasal meatus, and both ends advanced rostrally through the nostrils. To position a loop of obstetrical wire for the caudal septal incision, the ends of a loop of wire, each ensheathed with a catheter, were passed into the nasal cavity through the trephine portal—one end on the right side of the septum and the other on the left side of the septum. The ends were advanced into the nasopharynx, retrieved through the laryngotomy, and placed through a portion of a 12‐Fr thoracic drain tube, which was slid along the strands of the loop until the end of the tube resided within the nasopharynx.

After the three loops were positioned, the horse's head was examined radiographically to ensure no loop was entangled with another loop (Figure 2). The dorsal and ventral septal incisions were made in a caudal‐to‐rostral direction, and the caudal septal incision was made in a dorsorostral‐to‐ventrocaudal direction, through the laryngotomy, with the horse's head extended. All incisions were made simultaneously, each by a different operator. The dorsal and ventral incisions were connected rostrally with a scalpel when they were within 1–2 cm of the rostral border of the septum, thereby leaving a short rostral remnant of septum to support the soft tissues of the nares. The septum was grasped with large Vulsellum forceps and removed through a nostril. The nasal cavity was packed tightly with rolled gauze to control haemorrhage, and each nostril was temporarily occluded with a large horizontal‐mattress suture to retain the packing. The incision over the trephine portal was closed with skin staples. The laryngotomy and temporary tracheostomy were left unsutured.

**FIGURE 2:**
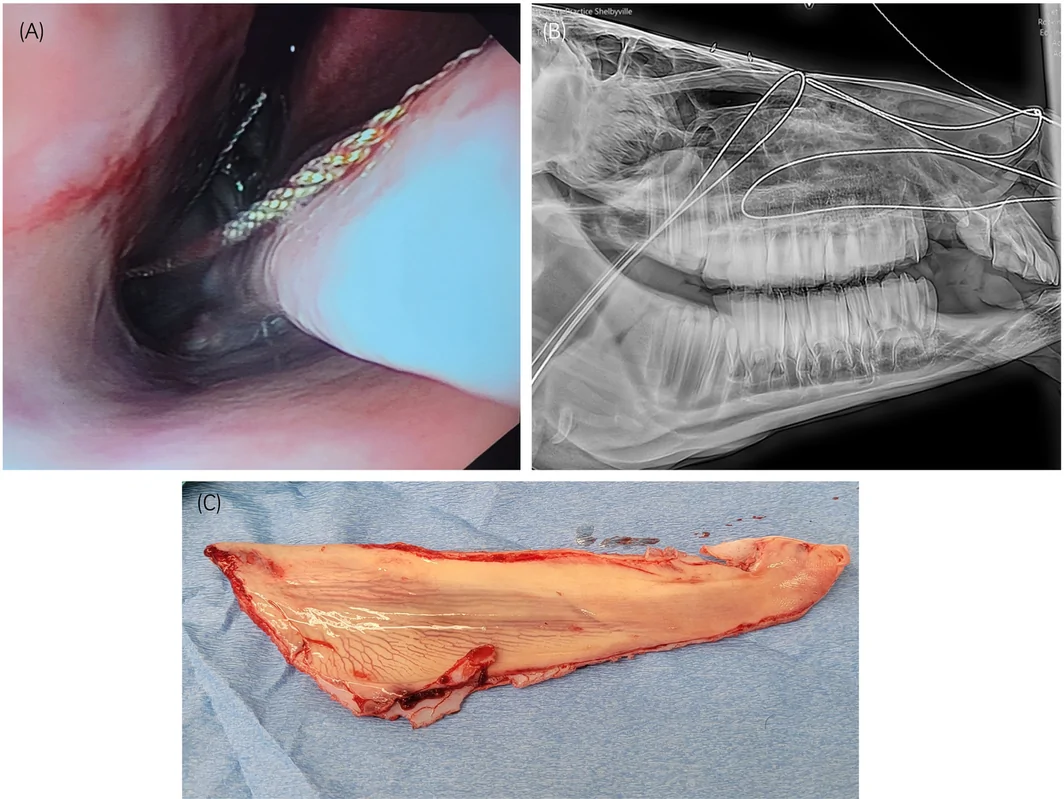
Endoscopic‐ and radiographic‐guided placement of obstetrical wires for excising the nasal septum of a weanling. (A) Endoscopic view during transnasal placement of an obstetrical wire through a nasal passage. (B) Lateral radiograph of the weanling's skull showing the three wires in position immediately before excising the nasal septum. (C) Resected nasal septum from a 7‐month‐old weanling.

#### 2.5.4 Transection, realignment, and fixation of the nasal bones

With the horse remaining in lateral recumbency and the concave side of the deformity uppermost, the dorsum of the nose rostral to the trephine portal was prepared for aseptic surgery. A 6‐ to 10‐cm cutaneous and subcutaneous incision, centred over the point of maximal curvature of the nasal bones, was made on dorsal midline that followed the curvature of the deformed nasal bones. The periosteum of each nasal bone was incised along the midline of the bone and elevated with a periosteal elevator (Figure 3A). The nasal bones and underlying parietal cartilage were transected perpendicular to their long axis at or just caudal to the point of maximal curvature using an oscillating bone saw (Figure 3B).

**FIGURE 3:**
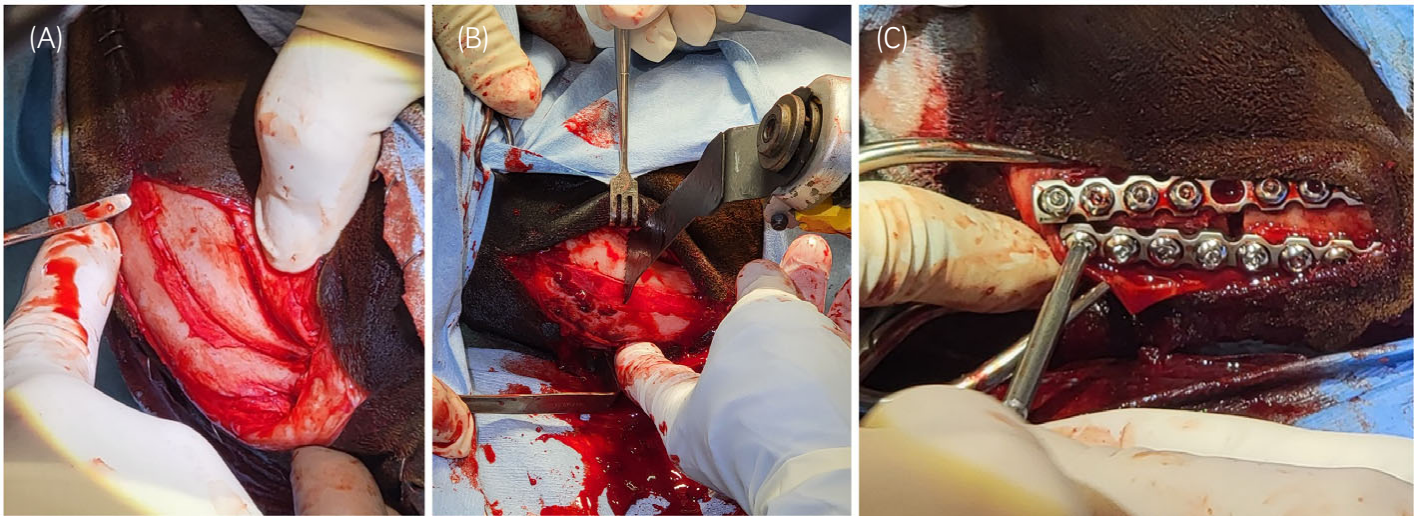
Transection, realignment, and fixation of the nasal bones during single‐stage correction of wry nose. (A) Exposure of the nasal bones at the point of maximal curvature. (B) Transection of the nasal bones perpendicular to their long axis at, or just caudal to, the point of maximal curvature using an oscillating bone saw. (C) Realignment of the transected nasal bones to the sagittal plane and stabilisation of the bones with contoured 2.7‐ or 3.5‐mm reconstruction or dynamic compression plates secured with cortical screws.

The transected nasal bones were rotated to the sagittal plane, creating a wedge‐shaped gap on the concave side of the deformity. This gap was closed by removing a wedge of bone of similar size from the nasal bone on the convex side of the deformity. Each transected nasal bone was fixed to its parent bone with a 2.7‐ or 3.5‐mm reconstruction or dynamic compression plate contoured to the dorsal surface of the bones and secured with 6‐ or 8‐mm cortical screws placed through the inner and outer laminae of the nasal bone (Figure 3C). The subcutaneous incision was closed with USP 2‐0 PDS placed in a simple‐continuous pattern, and the skin was closed with skin staples.

#### 2.5.5 Transection, realignment and fixation of the premaxillae and maxillae

To correct the deviation of the premaxillae, the horse was repositioned in dorsal recumbency. A dental wedge or small wooden block was placed between the cheek teeth to hold the mouth open, and the upper lip was retracted and secured to the skin of the bridge of the nose with towel clamps to expose the premaxillary incisors. The rostral aspect of the oral cavity was cleaned with an antiseptic solution.

A 3‐ to 5‐cm longitudinal gingival incision was made along the ventral border of the premaxillae at each interdental space, centred over the point of greatest curvature of the deformed premaxillae. The incision was extended through the periosteum, which was then elevated from the medial and lateral surfaces of the underlying bone (Figure 4A). The premaxillae and palatine processes were transected transversely at the point of greatest curvature using an oscillating bone saw and osteotome, taking care not to enter the nasal cavity (Figure 4B).

**FIGURE 4:**
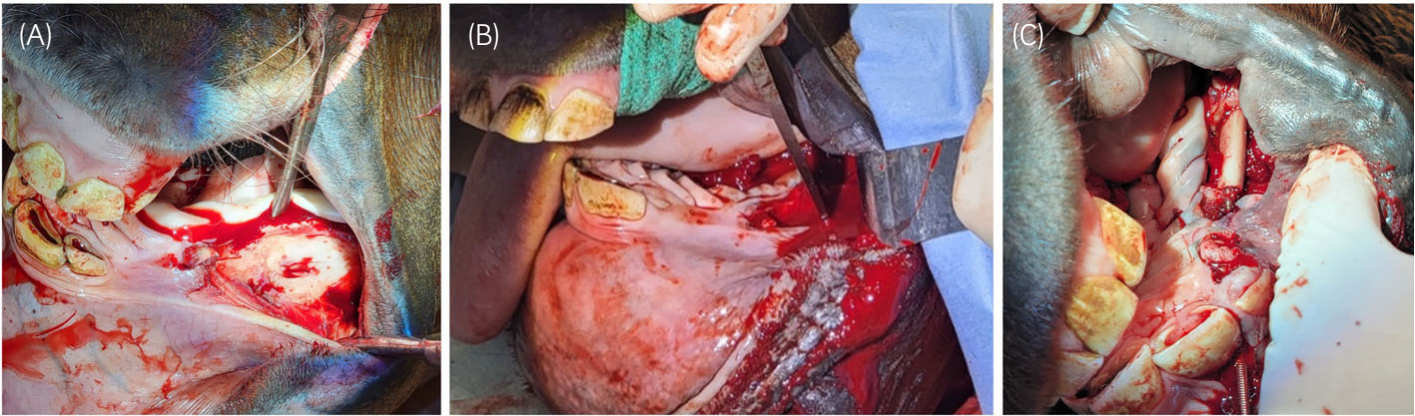
Intraoperative photographs illustrating transection, realignment, and stabilisation of the premaxillae during correction of wry nose deformity. (A) Exposure of the premaxillae following gingival incision and periosteal elevation. (B) Transverse osteotomy of the premaxillae and palatine processes using an osteotome. (C) Rib graft placement within the osteotomy gap following realignment, stabilised with transosseous pins and Kirschner wires.

After removing the wedge from between the cheek teeth, the transected segment of premaxillae was grasped with bone‐holding forceps and rotated toward the sagittal plane until the premaxillary and mandibular incisors were aligned. The premaxillary incisors were temporarily wired to the mandibular incisors to maintain alignment during fixation. The premaxillae were stabilised to the maxillae with a large Steinmann pin (4–6 mm diameter) placed through the premaxilla of the convex side of the deformity into the ipsilateral maxilla. Two to four Kirschner wires were placed through the premaxilla of the concave side of the deformity into the ipsilateral maxilla to improve rotational stability and, when a rib graft was placed longitudinally into the gap created by rotating the premaxillae, to secure the graft within the gap (Figure 4C). The insertion of the pin and wires was guided by using radiography (Figure 5). The exposed ends of pins and wires were cut close to the gingiva and driven beneath the mucosal surface with a punch and mallet. The gingival incisions in the interdental spaces were closed with barbed USP 0 PDS suture placed in a simple‐continuous pattern.

**FIGURE 5:**
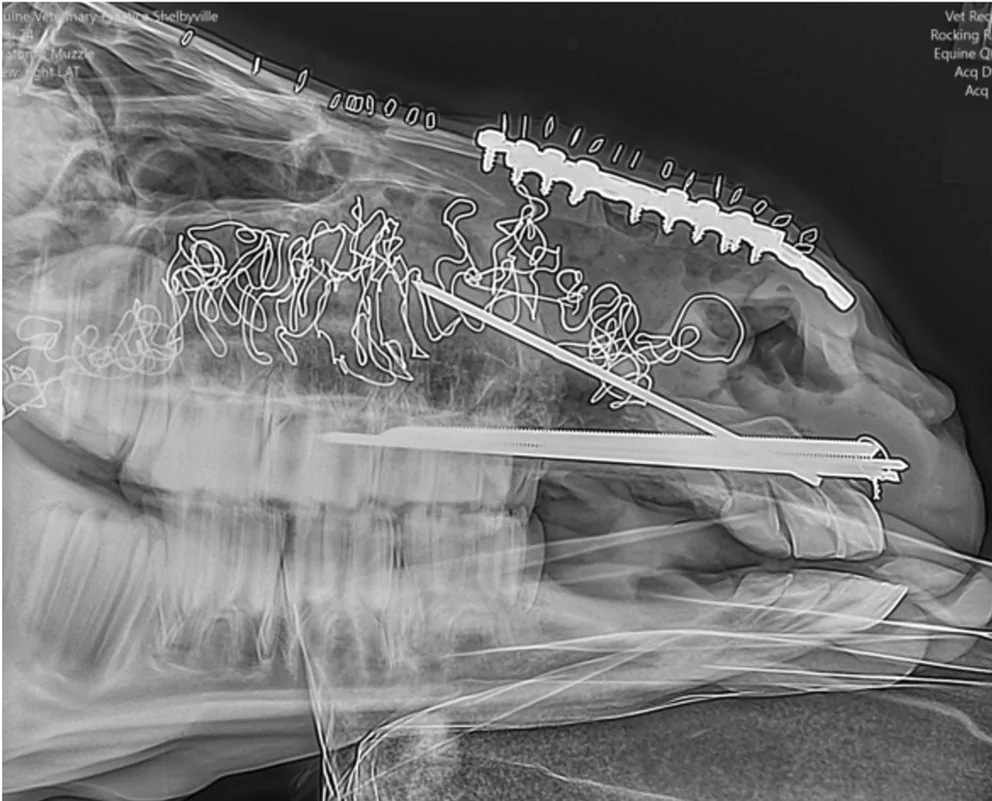
Postoperative laterolateral radiograph of the skull following surgical correction of a wry nose, showing alignment of the nasal bones and premaxillae. The nasal bones are stabilised with contoured reconstruction plates and cortical screws placed along the dorsal aspect, and the premaxillae are stabilised with transfixation pins. Extensive nasal packing is present within the nasal cavity, with the radiopaque thread of the rolled gauze visible radiographically.

### 2.6 Immediate postoperative management

The endotracheal tube was replaced with a temporary tracheostomy tube prior to recovery from anaesthesia, and the tube remained in situ for 2 to 3 days after surgery. Packing in the nasal cavity was removed on the day after surgery, and skin staples were removed at 12 to 14 days postoperatively.

The horses received potassium penicillin G (22,000 IU/kg, IV q6h; Penicillin G Potassium for Injection, USP; WG Critical Care, LLC), gentamicin (6.6 mg/kg, IV q24h; Gentamicin Injection, USP; Akorn Pharmaceuticals), and flunixin meglumine (1.1 mg/kg, IV q12h; Banamine®, Merck Animal Health) for 7–14 days. Butorphanol tartrate (0.02–0.05 mg/kg IV q2–4h as needed; Torbugesic®, Zoetis) was administered for additional analgesia during the first 1–3 postoperative days.

After recovering from general anaesthesia, the horses were fed a softened, complete pelleted ration for several days before being reintroduced to hay and grass pasture.

### 2.7 Alar cartilage fold resection

The alar fold of the concave side of the deformity was excised at the time implants were removed with the horse anaesthetised and in lateral recumbency, when indicated by persistent abnormal respiratory noise attributed to dynamic collapse of the alar fold and alar cartilage of that side of the deformity. The fold was exposed by splitting the nasal ala with an incision that extended from the nasoincisive notch through the margin of the nostril.

The alar fold was excised using scissors, and the mucosal margin of the wound was sutured to the cutaneous margin of the wound with USP no. 2‐0 PDS suture placed in a simple‐continuous pattern. The internal cutaneous layer of the lateral ala was closed using USP no. 2‐0 PDS suture placed in a simple‐continuous pattern, and the external cutaneous layer was closed with USP no. 2‐0 polypropylene suture placed in a vertical‐mattress pattern.

## 3 RESULTS

Four weanlings were eligible for inclusion in the study. All were fillies; three were American Quarter Horses, and one was an Arabian. The age of the weanlings at the time of surgery ranged from 6 to 8 months (median 7 months). All weanlings had difficulty breathing at presentation (4/4). Three weanlings were able to nurse and graze (3/4), whereas one could neither nurse nor graze (1/4). The severity of nasal deviation of two weanlings (2/4) was graded as very severe and that of two weanlings (2/4) as severe, using a grading system developed by Vasco et al.^14^ to describe the severity of deviation of the nose of horses affected with wry nose.

The transected premaxillae of all weanlings were stabilised by using a Steinmann pin and Kirschner wires (4/4), and the transected nasal bones of all foals were stabilised with plates and screws (4/4). One weanling (1/4) received a rib graft. Two weanlings (2/4) underwent resection of the alar fold on the concave side of the deformity. The pin and wires were removed 6–19 weeks postoperatively (median 8.5 weeks), and the plates/screws were removed from the nasal bones 8–19 weeks postoperatively (median 12 weeks).

All horses (4/4) developed infection around the implanted bone plates and screws. The sutured gingival incisions of three horses (3/4) dehisced. The pin and wires of one horse (1/4) were extruded prematurely. One horse (1/4) suffered partial collapse of tracheal rings at the site of a temporary tracheostomy. One (1/4) developed an oronasal fistula, and one (1/4) was left postoperatively with a class 3 malocclusion.

At follow‐up, three horses (3/4) were reported to have good respiratory capacity at rest and during performance and made no abnormal respiratory noise at rest or at exercise. One (1/4) was reported to have poor respiratory capacity during performance and made an abnormal respiratory noise at rest and at exercise.

All horses (4/4) were reported to graze and masticate normally and were able to maintain good body condition. Only one horse (1/4) was reported to require more than routine dental care. Three horses (3/4) were reported to be able to perform their intended athletic endeavour. One owner was uncertain if his or her foal would be able to perform as an athlete. All owners (4/4) reported they were satisfied with their horse's functional and cosmetic outcome. Three owners (3/4) were very satisfied with their horse's outcome; one (1/4) owner expressed ‘neutral’ satisfaction.

## 4 DISCUSSION

Surgical correction of wry nose of the four weanlings described in this report resulted in a good functional outcome. Surgery provided most horses with good respiratory capacity at rest and during exercise, and all horses were able to graze and masticate normally. All four horses had severe to very severe facial deformity, and all were reported to have respiratory compromise preoperatively. The most severely affected weanling was unable to nurse or graze preoperatively and required more than routine attention to its dentition. Owner‐ and surgeon‐reported outcomes generally supported a favourable clinical outcome, although one horse had persistent abnormal respiratory noise at rest and exercise and reduced respiratory capacity during activity.

Only one foal, the most severely affected, received a rib graft to span the gap that resulted on the concave side of the deformity when the premaxillae were repositioned. Reports of the surgical techniques used to correct wry nose describe inserting a bone graft (i.e., a section of rib) into the gap to provide structural support and an osteoconductive scaffold to speed healing.^1^, ^2^, ^5^ Authors of more recent reports, however, found that stable fixation allows formation of a bridging callus and union.^3^, ^6^ The transected premaxillae of the three foals described in this report that did not receive a rib graft healed without complication, despite the presence of a postoperative gap between bony segments, supporting the observation that inserting a bone graft may not be necessary when alignment is acceptable and the transected premaxillae are stable. Though insertion of a rib graft may moderately increase the stability of the construct, harvesting a segment of rib and stabilising it within the gap created on the concave side of the deformity increases the time of surgery substantially.

Cousty^3^ reported that a 12‐mm gap on the concave side of the deformity of a yearling with wry nose that resulted when the premaxillae were repositioned healed without complication when the transected premaxillae were stabilised with an external fixator. Similarly, Sapper and colleagues^6^ reported good bone healing of two foals undergoing correction of wry nose even though a bone graft was not inserted into the gap created by repositioning the premaxillae. Based on our experience and that of others,^3^, ^6^ we believe that inserting a bone graft into the gap created by repositioning the premaxillae of a horse suffering from wry nose is unnecessary, provided fixation of the transected premaxillae is adequate.^2^, ^6^

In a report of 13 horses that underwent surgery to correct wry nose, it was noted that operative time can be reduced when one surgical team harvests the rib graft while another excises the nasal septum or performs the transection and fixation of the nasal bones.^13^ Omitting the harvest of a rib graft allows the surgery to correct wry nose to be completed more rapidly or with fewer personnel and eliminates the possibility of a complication associated with harvesting the rib.

All four foals of our study developed implant‐associated infection at the site of transection of the nasal bones, necessitating removal of the plates and screws after the site of transection had healed. Penetration of the screws into the nasal cavity, with subsequent contamination of the site of implantation, is the most likely cause of the implant‐associated infection. Each transected nasal bone of each weanling was fixed to its parent bone with a 2.7‐ or 3.5‐mm plate and secured with 6‐ or 8‐mm cortical screws placed through the nasal bone. The authors have observed that screws of this length are likely to penetrate into the nasal cavity, especially when placed through the thin nasal bones of weanlings, resulting in bacterial contamination of the surgical site. Infection at the site of implantation at the nasal bones occurred less frequently in other reports describing the results of surgery to correct wry nose.^2^, ^11^ Schumacher's team^2^ reported that one of three horses developed swelling and two draining tracts over the nasal bones at the site of plate‐and‐screw fixation. The swelling and drainage resolved after the implants were removed 18 months postoperatively. In another report, 3 of 13 horses developed infection of the soft tissues around the plates and screws used to transfix the nasal bones 4–18 months after surgery, necessitating removal of the implants from two of these three horses, whereas in the third horse the implants were shed spontaneously.^13^

When securing the plates to the nasal bones, the shortest screws available that engage both cortices of the nasal bones should be used to reduce the risk of penetrating the nasal mucosa. When infection is recognised at the site of implantation, exudate should be cultured for bacteria, and antimicrobial treatment should be based on antimicrobial sensitivity testing of bacteria identified. The implants should be removed when bony stability is achieved.

The sutured gingival incisions, at the site at which the premaxillae were transected, of 3 of the 4 weanlings of this report, dehisced, resulting for one of these 3 weanlings in an oronasal fistula (Figure 6), most likely because the nasal mucosa was penetrated during the premaxillary osteotomy. Dehiscence of gingival incisions of horses is common^7^ and results from tension, compromised blood supply, and contamination.^4^, ^5^, ^6^, ^7^ Despite partial or complete dehiscence of the gingival incisions of these three foals, the gingival wounds healed by second intention without compromising stability of the premaxillary osteotomies. The fistula healed by second intention by approximately 50 days after surgery after repeated debridement and packing of the defect.

**FIGURE 6:**
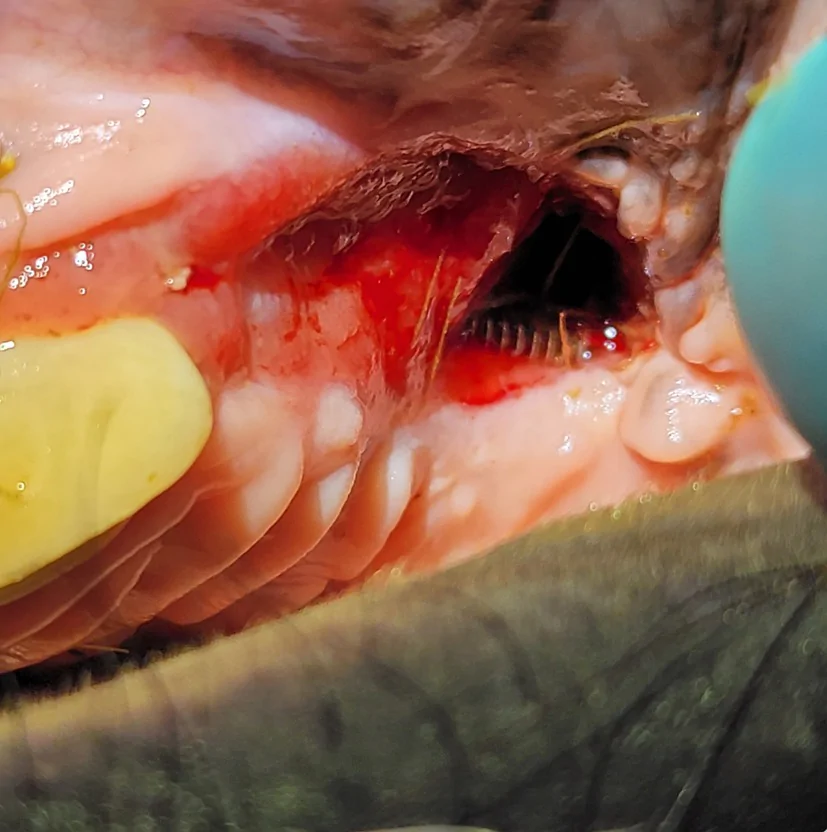
Oral photograph of an oronasal fistula on the concave side of the deviation 9 days postoperatively.

Premature extrusion of the Steinmann pin and Kirschner wires used to stabilise the transected premaxillae, as was observed in one horse (1/4) in this report, has been reported by others to be a complication of surgical correction of wry nose.^1^, ^7^, ^13^ The Steinmann pin and Kirschner wires were removed electively from the other three horses 6–9 weeks after repair, when the premaxillae were stable.

One foal developed collapse of two tracheal rings at the site of temporary tracheostomy. Complications (e.g., pressure necrosis, formation of exuberant granulation tissue, stenosis) related to a temporary tracheostomy of horses are uncommon.^20^ Collapse of the tracheal rings may have occurred because of excessive disruption of the annular ligament (i.e., >180°) for insertion of the endotracheal tube for administration of an inhalant anaesthetic, combined with sustained pressure from a rigid, self‐retaining metal tracheostomy tube, resulting in weakening and deformation of the tracheal rings. Stenosis resulting from deformity of one or more tracheal rings is described as a rare complication of temporary tracheostomy.^21^, ^22^ Increase in the size of the tracheal lumen at the site of stenosis, as the foal grew, resulted in resolution of the respiratory compromise.

One foal in this report was left postoperatively with its mandibular incisors positioned rostral to the maxillary incisors [i.e., a class 3 malocclusion (MAL3)].^22^ This is a recognised sequela to correction of wry nose because affected foals often have foreshortened premaxillae.^7^ In addition, disruption or resection of the nasal septum may further retard growth of the maxillae. It has previously been reported that three foals treated for wry nose by using an external fixator to stabilise the transected premaxillae experienced shortening of the premaxillae/maxillae at a one‐year follow‐up.^4^ Persistent brachygnathia superior and incisor malocclusion after correction have also been reported previously^2^, ^6^ demonstrating the risk of long‐term occlusal abnormalities that may result in the horse requiring specialised dental care.

Two weanlings underwent resection of the alar fold on the concave side of the deformity to resolve the potential for dynamic narrowing of the nasal vestibule resulting from collapse of the alar fold, which has been associated with deviation or thickening of the nasal septum.^23^ Abnormal respiratory noise after correction of wry nose may result from turbulence of air flowing through the nasal cavity after excision of the nasal septum,^3^, ^4^, ^7^, ^13^, ^17^ and collapse of one or both alar folds may further contribute to the abnormal noise. Abnormal respiratory noise following correction of wry nose was eliminated from two of three horses after the alar folds were resected,^13^ supporting its use as an adjunctive procedure when collapse of the alar folds, as a cause of abnormal respiratory noise, is suspected. Unaddressed collapse of the alar folds of the horse in our study reported to have persistent abnormal respiratory noise at rest and during exercise and reduced respiratory capacity during exercise may have contributed to the abnormal noise and exercise intolerance.

All 4 weanlings of this report were experiencing respiratory difficulty when they were presented, resulting from restriction of the airflow through the nasal cavity. Each owner reported that the respiratory function of his or her horse improved after the facial deviation was corrected, but abnormal respiratory noise persisted, and one horse was reported to have reduced respiratory capacity during exercise. The long‐term outcome was favourable from the perspective of each owner; three horses were reported to be able to perform their intended athletic function, and all were reported able to maintain good body condition and graze and masticate normally. Overall, owners were satisfied with the functional and cosmetic outcome, even if his or her horse made an abnormal respiratory noise or had less than optimal respiratory capacity. All owners perceived his or her horse's quality of life and appearance to be improved (Figure 7).

**FIGURE 7:**
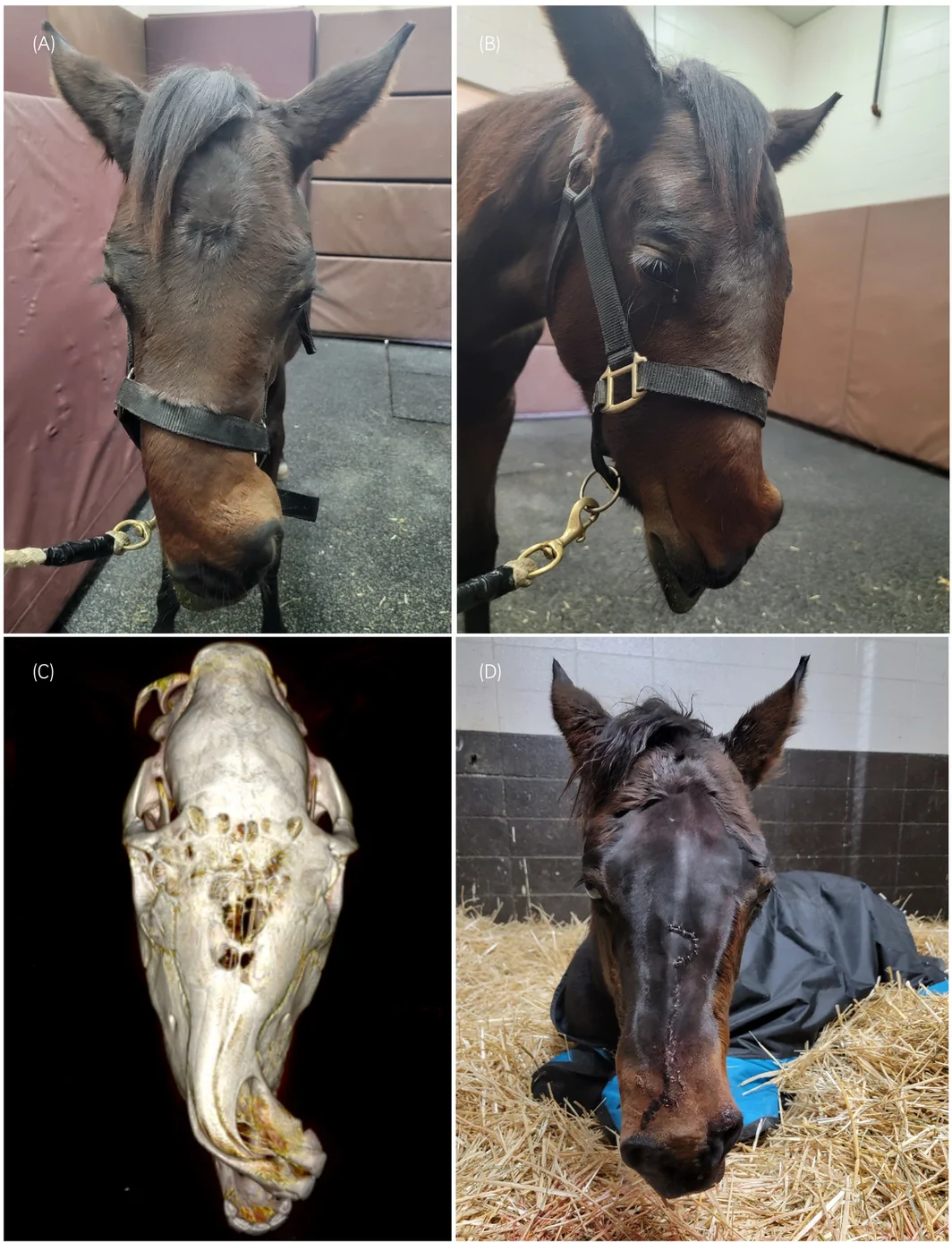
Single‐stage orthognathic correction of wry nose in a 7‐month‐old weanling using a transection‐and‐fixation technique. (A) A preoperative frontal view showing marked lateral deviation of the maxillae/premaxillae. (B) Preoperative lateral view demonstrating deviation and asymmetry of the rostral aspect of the face. (C) Preoperative three‐dimensional CT reconstruction (dorsal view) illustrating facial deformity and malalignment of the rostral facial bones. (D) Postoperative frontal view following single‐stage orthognathic correction, showing improved alignment and appearance of the rostral aspect of the face after transection and fixation.

## 5 CONCLUSION

Single‐stage correction of campylorrhinus lateralis using a transection‐and‐fixation technique resulted in satisfactory long‐term functional and cosmetic outcomes in this small group of horses. All horses were reported to graze and masticate normally, and owners were satisfied with the overall result. Respiratory function of all horses improved, and three horses were reported to be able to perform their intended athletic endeavour, although one of these three horses had reduced respiratory capacity. Postoperative complications were common, particularly implant‐associated infection at the site at which the transected nasal bones were stabilised. Adjunctive procedures, such as alar fold resection, may be necessary to resolve or ameliorate abnormal respiratory noise after surgery. The owner should be thoroughly counselled before surgery to manage his or her expectations.

## FUNDING INFORMATION

Not applicable.

## CONFLICT OF INTEREST STATEMENT

The authors have declared no conflicting interests.

## AUTHOR CONTRIBUTIONS

**Helio Vasco:** Conceptualization; investigation; writing – original draft; methodology; data curation; visualization. **Jim Schumacher:** Conceptualization; investigation; writing – review and editing; methodology; supervision; resources; visualization; data curation. **Jack Easley:** Conceptualization; investigation; writing – review and editing; methodology; supervision; resources; data curation.

## DATA INTEGRITY STATEMENT

Helio Vasco, Jim Schumacher, and Jack Easley had full access to all the data in the study and take responsibility for the integrity of the data and the accuracy of data analysis.

## ETHICAL ANIMAL RESEARCH

Research ethics committee oversight not required by this journal: retrospective study of clinical records.

## INFORMED CONSENT

Explicit owner consent for inclusion of animals in this study was not obtained. Owners or their agents were made aware that case information may be used for research in general.

## Data Availability

The data that support the findings of this study are available upon reasonable request from the corresponding author. Open data sharing exemption granted by the editor.
